# Melatonin regulates mitochondrial dynamics and alleviates neuron damage in prion diseases

**DOI:** 10.18632/aging.103328

**Published:** 2020-06-10

**Authors:** Xixi Zhang, Deming Zhao, Wei Wu, Syed Zahid Ali Shah, Mengyu Lai, Dongming Yang, Jie Li, Zhiling Guan, Wen Li, Hongli Gao, Huafen Zhao, Xiangmei Zhou, Lifeng Yang

**Affiliations:** 1 Key Laboratory of Animal Epidemiology and Zoonosis, Ministry of Agriculture, National Animal Transmissible Spongiform Encephalopathy Laboratory, College of Veterinary Medicine, China Agricultural University, Beijing 100193, China; 2 Department of Pathology, Faculty of Veterinary Sciences, Cholistan University of Veterinary and Animal Sciences, Bahawalpur 63100, Pakistan

**Keywords:** prion disease, melatonin, mitochondrial dynamics, apoptosis

## Abstract

Prion diseases are neurodegenerative diseases associated with neuron damage and behavioral disorders in animals and humans. Melatonin is a potent antioxidant and is used to treat a variety of diseases. We investigated the neuroprotective effect of melatonin on prion-induced damage in N2a cells. N2a cells were pretreated with 10 μM melatonin for 1 hour followed by incubation with 100 μM PrP^106-126^ for 24 hours. Melatonin markedly alleviated PrP^106-126^-induced apoptosis of N2a cells, and inhibited PrP^106-126^-induced mitochondrial abnormality and dysfunction, including mitochondrial fragmentation and overproduction of reactive oxygen species (ROS), suppression of ATP, reduced mitochondrial membrane potential (MMP), and altered mitochondrial dynamic proteins dynamin-related protein 1 (DRP1) and optic atrophy protein 1 (OPA1). Our findings identify that pretreatment with melatonin prevents the deleterious effects of PrPSc on mitochondrial function and dynamics, protects synapses and alleviates neuron damage. Melatonin could be a novel and effective medication in the therapy of prion diseases.

## INTRODUCTION

Prion diseases are a group of fatal neurodegenerative disorders characterized by loss of motor control, paralysis, wasting and eventual death [1, 2]. Prion diseases are generally referred to as transmissible spongiform encephalopathy (TSE) because they can be transmitted from one host to another and cause the histological appearance of large vacuoles in the cortex and cerebellum. In many neurodegenerative diseases, synapse loss is a common pathological change [3, 4]. Synapses are contact points between two neurons, at which neurons communicate by passing ions or neurotransmitters the synaptic cleft. Synaptic integrity is crucial for effective neuronal communication.

Mitochondria are important organelles in all cell types, but they are particularly critical in the nervous system. The study of mitochondria is crucial to understanding neurodegenerative diseases. The proper functioning of dynamic mitochondrial processes is essential to neuronal processes and communication [5]. Mitochondrial dynamic processes include the movement of mitochondria along the cytoskeleton, the regulation of mitochondrial architecture (morphology and distribution), and connectivity mediated by tethering and fusion/fission events [6]. Abnormalities in mitochondrial fusion and fission are involved in many injury processes in various systems of the human and animal body, including optic atrophy, ischemia-reperfusion injury, and neurodegenerative diseases [6–8].

Melatonin is produced by the pineal gland and has potent antioxidant activities. Several lines of evidence indicate that melatonin protects mitochondrial, which could prevent the development and progression of neurodegeneration [9, 10]. Melatonin treatment provided beneficial effects in an Alzheimer model related to tauopathy by improving the autophagic flux and, thereby, preventing cognitive decline [11]. The antioxidant activity and mitochondrial protection of melatonin were considered to be responsible for its neuroprotective effects against amphetamine-induced toxicity to the hippocampus, the primary brain area involved in learning and memory process, in neonatal rats [12]. Melatonin was also shown to protect against the neurotoxicity of cadmium by maintaining the balance between mitochondrial fusion and fission [13]. However, the effect of melatonin on mitochondrial protection of neurons from prion is unknown.

Therefore, we demonstrated that pretreatment with melatonin prevented PrP^106-126^ induced neuron damage by maintaining synapse and mitochondria functions and mitochondria dynamics in an *in vitro* prion model.

## RESULTS

### Melatonin attenuates PrP^106-126^ -induced N2a cell apoptosis

**Figure 1:**
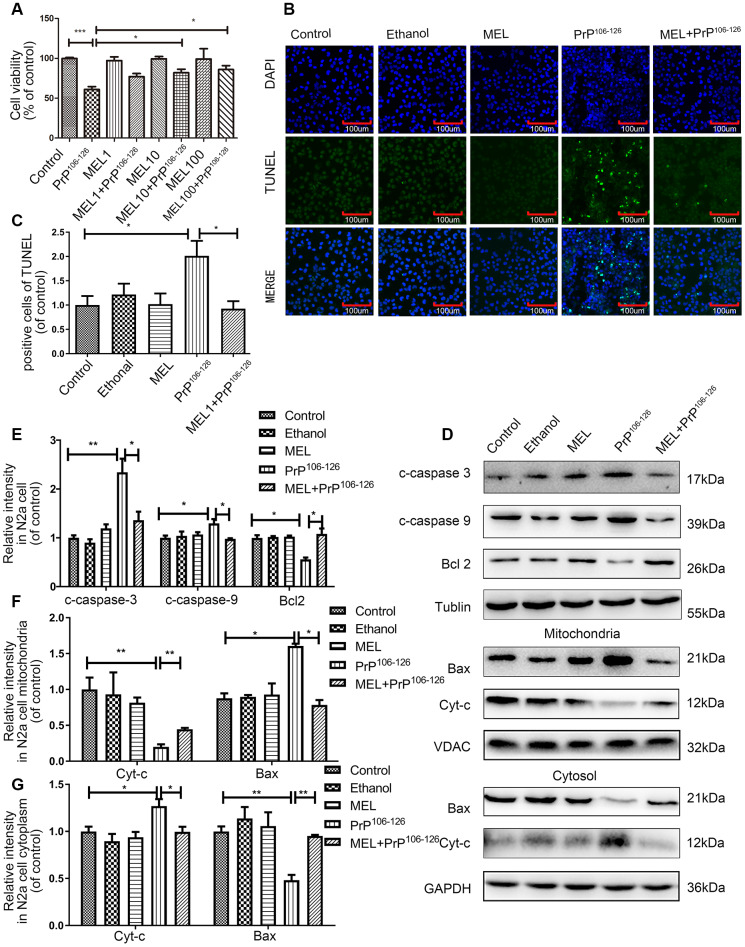
**Melatonin attenuated PrP^106-126^-induced N2a cell apoptosis.** (**A**) N2a cell viability was assayed using the CCK8 kit after treatment with melatonin and PrP^106-126^. (**B**, **C**) N2a cell apoptosis was assayed by TUNEL staining. (**D**–**G**) Protein expression of cleaved caspase-9, cleaved caspase-3 and Bcl2 in N2a cells, and protein expression of cytochrome c and Bax in cytosolic and mitochondrial extracts of N2a cells by western blotting. *P < 0.05, **P < 0.01, ***P < 0.001. All experiments were repeated at least three times.

To assess the protective effect of melatonin against PrP^106-126^-induced apoptosis of N2a cells, N2a cells were preincubated with 1, 10, or 100 μM of melatonin for 1 h before 100 μM PrP^106-126^ peptide was added and further incubated for 24 h. Exposure to 10 or 100 μM melatonin protected cells from PrP^106-126^ peptide-induced toxicity. Cell viability measured by the CCK8 assay was increased to 81.92-85.93% by 10 and 100 μM melatonin from 60.92% in the PrP^106-126^ control (Figure 1A). No cytotoxicity was observed at up to 100 μM melatonin. TUNEL assay showed that melatonin inhibited N2a cell apoptosis induced by the TSE peptide (Figure 1B and 1C). Additionally, exposure to melatonin decreased the abundance of cleaved caspase-3 and cleaved caspase-9, whereas the abundance of anti-apoptosis factor Bcl2 was increased. Separation of the cytosolic and mitochondrial extracts enabled us to determine the distribution of cytochrome c and Bax in mitochondria and cytosol. Melatonin reduced the release of cytochrome c from mitochondria, whereas the abundance of Bax was restored in the cytosol (Figure 1D–1G).

### Melatonin reduces synapse damage and restores mitochondrial distribution in PrP^106-126^-exposed N2a cells

**Figure 2:**
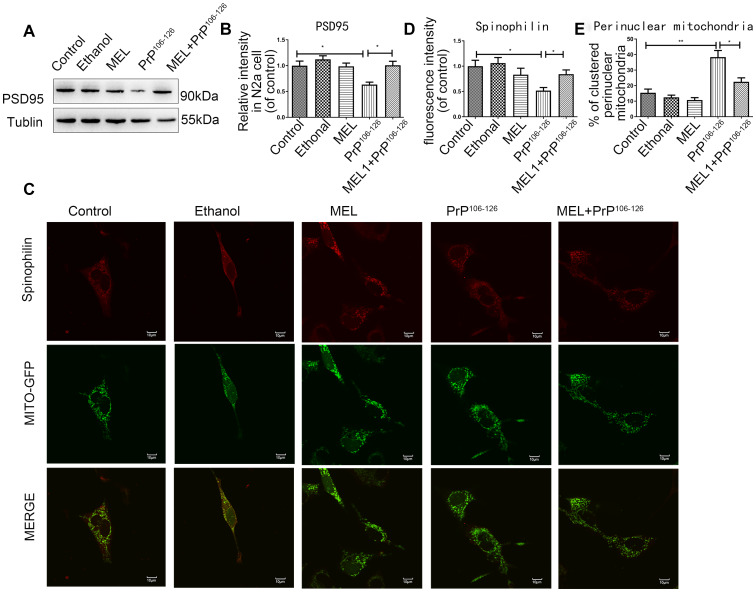
**Melatonin reduced synapse damage in PrP^106-126^-treated N2a cells.** (**A**, **B**) Protein expression of PSD95 by Western blotting. (**C**) Representative images of mitochondria (Original magnification 600×). (**D**) Quantification of spinophilin. (**E**) Cells with clustered perinuclear mitochondria *P < 0.05, **P < 0.01. All experiments were repeated at least three times.

Synapses are primary sites for information transmission between neurons, and intact synaptic morphology is critical in neuronal function [14, 15]. Postsynaptic density protein-95 (PSD95) is a scaffolding protein in the synapse and a regulator of synaptic strength. As shown in Figure 2A and 2B, PrP^106-126^ exposure reduced the abundance of PSD95 by 63.45%. Pretreatment with melatonin prevented the prion peptide-induced reduction of PSD95. Spinophilin is an actin- and protein phosphatase-1 (PP1) binding protein, which is specifically enriched in dendritic spines [16], and thus serves as a dendritic spine marker. Immunofluorescence staining of spinophilin showed that the abundance of dendritic spines in the cells treated with PrP^106-126^ was lower than that of the untreated control cells, but melatonin alleviated the inhibitory effect of PrP^106-126^ on spinophilin (Figure 2C, 2D). These observations suggest that pre-treatment with melatonin protected synapses from damage induced by PrP^106-126^. As shown in Figure 2C and 2E, PrP^106-126^ induced an uneven distribution of mitochondria in N2a cells, with mitochondria clustered around the nucleus and decreased distribution in the axons. In contrast, cells pre-treated with melatonin prior to PrP^106-126^ treatment showed a relatively uniform distribution of mitochondria.

### Melatonin ameliorates PrP^106-126^-induced mitochondrial fragmentation in N2a cells

**Figure 3:**
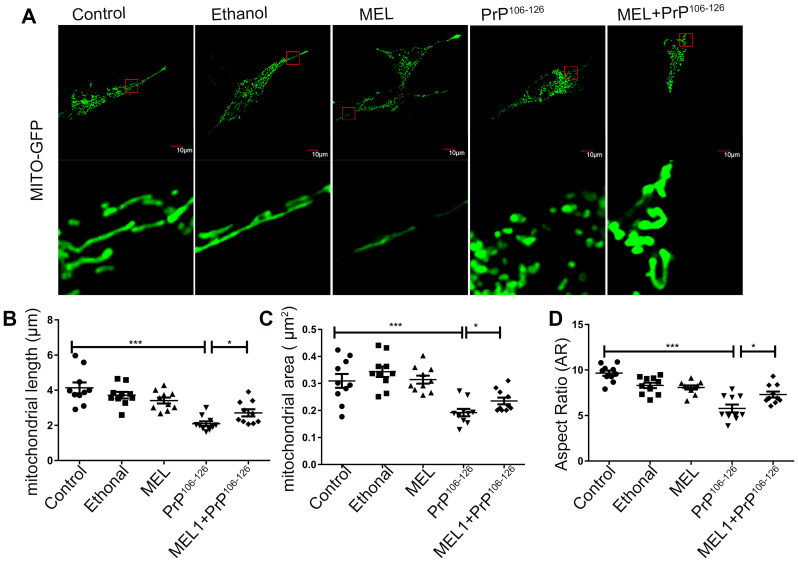
**Melatonin ameliorated PrP^106-126^-induced mitochondrial fragmentation in N2a cells.** Mitochondrial morphology was detected by confocal microscopy (**A**) and mitochondrial length (**B**), area (**C**) and aspect ratio (AR) (**D**) were analyzed by the ImageJ software. *P < 0.05, ***P < 0.001. All experiments were repeated at least three times.

Mitochondria-related apoptosis and mitochondrial damage are common features of neurodegenerative diseases [17]. Mitochondria in untreated control neurons are generally in tubular form. In PrP^106-126^–treated cells, mitochondria displayed round, punctate-like fragments, while mitochondria in the cells pre-treated with melatonin showed tubular patterns similar to the untreated control (Figure 3A). The average length of the mitochondria of cells pre-treated with melatonin was significantly longer than that of the mitochondria of cells treated with PrP^106-126^ alone (2.01μm *cf.* 4.13 μm) (Figure 3B). Mitochondrial aspect ratio (AR) and area markedly increased in cells pre-treated with melatonin in comparison with cells treated with PrP^106-126^ alone (Figure 3C, 3D). These results demonstrated that pre-treatment with melatonin protected mitochondrial morphology of N2a cells from damage by PrP^106-126^.

### Melatonin protects neuron cells from PrP^106-126^-induced mitochondrial dysfunction

**Figure 4:**
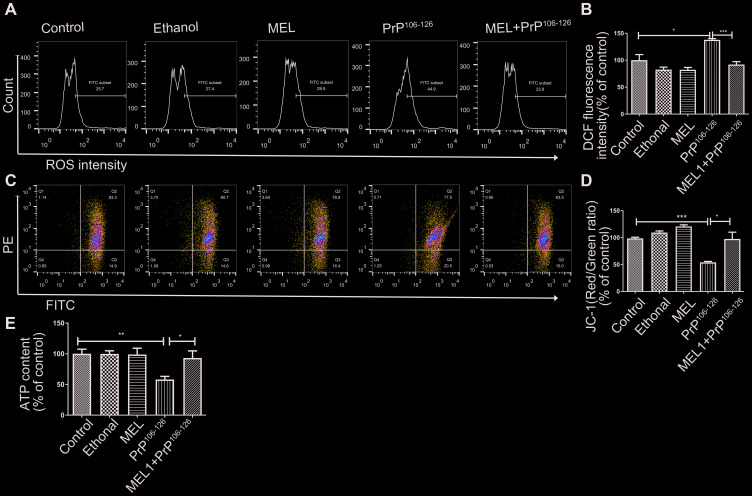
**Melatonin protected N2a cells from PrP^106-126^-induced mitochondrial dysfunction.** Fluorescence was detected by flow cytometry (FACS) analysis of ROS production (**A**, **B**) and JC-1 as a marker of mitochondrial membrane potential (MMP) (**C**, **D**) in N2a cells after treatment. The horizontal axis shows the FITC. (**E**) ATP levels. *P < 0.05, **P < 0.01, ***P < 0.001. All experiments were repeated at least three times.

After examining the mitochondrial morphology, we examined the effect of melatonin on mitochondrial function. Compared with the untreated control group, cells treated with PrP^106-126^ showed elevated ROS production, while cells pre-treated with melatonin showed ROS production similar to levels of the untreated control (Figure 4A, 4B). The MMP of the cells treated with PrP^106-126^ alone was 53.25% of that of the control cells, and the effect of the prion peptide on MMP was completely reversed by melatonin (Figure 4C, 4D). The ATP level of the cells pre-treated with melatonin was also higher than that of the cells treated with PrP^106-126^ alone (Figure 4E). These results showed that melatonin attenuated PrP^106-126^-induced mitochondrial dysfunction by inhibiting ROS overproduction, restoring the MMP, and increasing ATP production.

### Melatonin regulates DRP1 and OPA1 in cells with PrP^106-126^-induced disruption of mitochondrial dynamics

**Figure 5:**
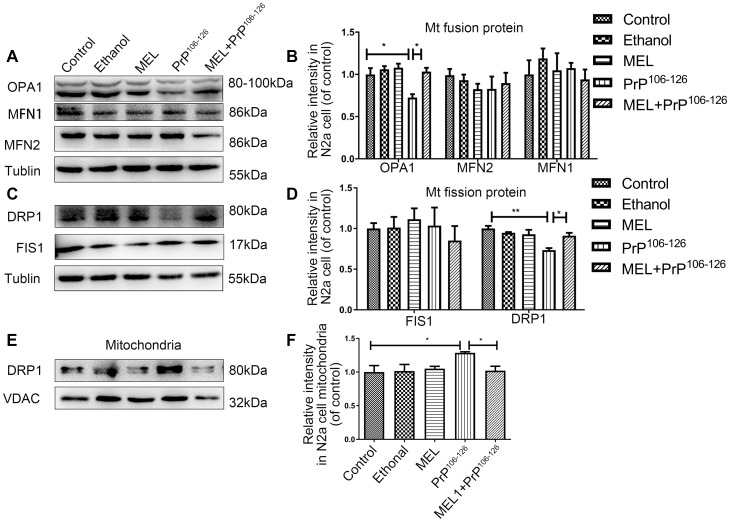
**Melatonin completely prevented the effect of PrP^106-126^ on the protein expression of DRP1 and OPA1.** Mitochondrial fusion proteins (MFN1, MFN2, and OPA1) (**A**, **B**) and mitochondrial fission proteins (DRP1 and FIS1) (**C**, **D**) in N2a cells and DRP1 in mitochondria (**E**, **F**) by Western blotting. *P < 0.05, **P < 0.01 All experiments were repeated at least three times.

Imbalance of mitochondrial dynamics occurs in neurodegenerative diseases [18]. Previous studies by our groups revealed that DRP1 [19] (a mitochondria fission protein) and OPA1 [20] (a mitochondria fusion protein) are pivotal in PrP^Sc^-associated mitochondria dysfunction and neuron apoptosis. To determine whether melatonin maintains mitochondrial dynamics and homeostasis, the expression levels of proteins involved in mitochondrial fusion and fission were measured. The protein expression of OPA1 was reduced after PrP^106-126^ treatment, and application of melatonin increased the protein expression to the untreated and uninfected control level (Figure 5A, 5B). Next, whole cell and mitochondrial levels of DRP1 were measured. PrP^106-126^ treatment resulted in a decrease in cellular DRP1 but an increase in mitochondrial DRP1, and the effects of PrP^106-126^ were prevented by melatonin (Figure 5C–5F). Fission1 (FIS1) and fusion protein mitofusin-1/2 (MFN1/2) remained unaffected by the prion peptide or melatonin.

### Melatonin and mitochondrial dynamic proteins regulate mitochondrial function in PrP^106-126^-induced prion models

**Figure 6:**
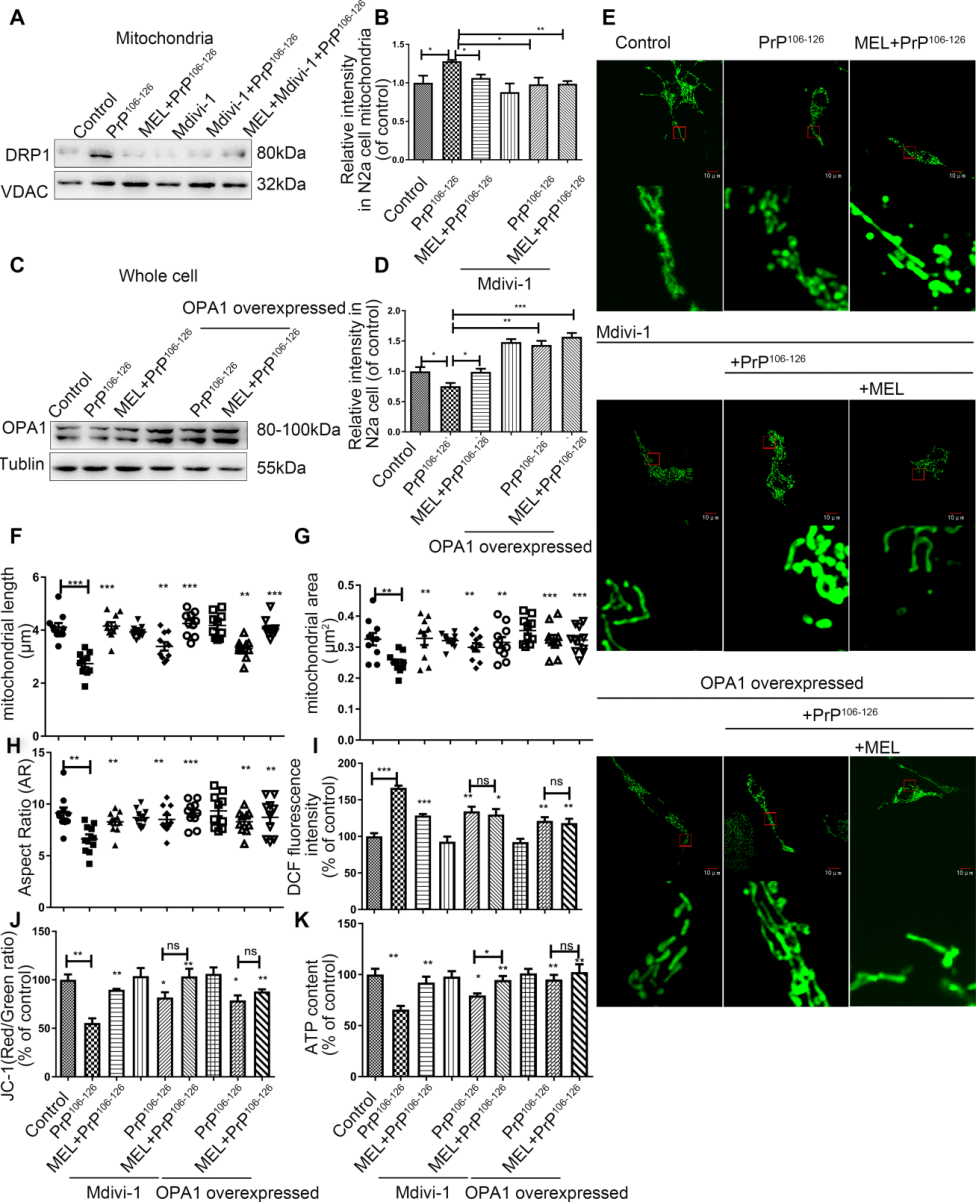
**Melatonin and mitochondrial dynamic proteins regulate mitochondrial function in PrP^106-126^-induced prion models.** (**A**, **B**) Protein levels of DRP1 in mitochondria by Western blotting. (**C**, **D**) Protein levels of OPA1 in whole cells by Western blotting. (**E**) Representative photomicrographs of mitochondria by confocal fluorescence microscopy showing mitochondrial morphology (Original magnification 600×). (**F**–**H**) Morphometric measurement of mitochondria. (**I**–**K**) Mitochondrial function - ROS production (**J**), mitochondrial membrane potential (MMP) (**K**) and ATP levels. *P < 0.05, **P < 0.01, ***P < 0.001, comparison with PrP^106-126^ group. All experiments were repeated at least three times.

To further investigate the role of melatonin and mitochondrial dynamic proteins in prion diseases, we measured DRP1 and OPA1 expression in N2A cells treated with PrP^106-126^ and a DRP1 inhibitor, Mdivi-1 and in N2a cells overexpressing OPA1. Similar to melatonin, Mdivi-1 (10 μM, concentration based on a published study [21]) inhibited PrP^106-126^-induced increase of DRP1 expression in mitochondria (Figure 6A, 6B). In N2a cells overexpressing OPA1, neither PrP^106-126^ nor melatonin affected OPA1 levels (Figure 6C, 6D).

We also studied mitochondria morphology and function after treatment with PrP^106-126^ and Mdivi-1 and in OPA1-overexpressing cells. Mdivi-1 treatment or overexpression of OPA1 protected mitochondria from fragmentation induced by the prion peptide as shown by IFA (Figure 6E) and morphometry assessment (mitochondrial length, area, and aspect ratio) (Figure 6F–6H). As shown in Figure 6I–6K, abnormal mitochondrial function induced by PrP^106-126^ was partially protected by DRP1 inhibition or OPA1 overexpression, including the abundance of ROS/ATP and MMP. Treatment with Mdivi-1 or overexpression of OPA1 exhibited similar protective effects against PrP^106-126^-induced mitochondrial dysfunction, suggesting that both DRP1 and OPA1 are critical in maintaining mitochondrial function. Moreover, combined treatment with Mdivi-1 and melatonin increased the abundance of ATP more effectively than treatment with Mdivi-1 alone (Figure 6K).

## DISCUSSION

Melatonin is an antioxidant molecule with a strong capacity to scavenge ROS and NOS [7] and is associated with ageing and multiple diseases, including neurodegenerative disorders [10, 22, 23]. The present study provides evidence that pretreatment with melatonin protected PrP^106-126^-treated N2a cells from synaptic and mitochondrial damage and could be utilized in the treatment of prion and other neurogenerative diseases.

PrP^106-126^ is widely used as a model for studying PrP^Sc^ neurotoxicity because it leads to neuronal apoptosis and cytotoxicity [24–26]. In our study, Bax accumulation in and the release of cytochrome c from mitochondria were observed, while the expression of Bcl-2, an anti-apoptotic factor, was decreased. There was also activation of caspase-9 and caspase-3, which participates in mitochondria-mediated apoptosis pathways [27, 28]. Our results suggest that mitochondrial damage is responsible for PrP^106-126^-induced neuronal apoptosis. Importantly, our study demonstrated that pretreatment with melatonin alleviated mitochondria-mediated apoptosis induced by PrP^106-126^. Using a transgenic mouse model of Alzheimer's disease (AD), Feng et al. also showed that melatonin treatment significantly down-regulated the expression of apoptosis-related factors [29].

Synapses, which act as functional links between neurons and are responsible for information transmission [30], are rapidly damaged during the development of prion diseases [31]. Synapse loss has detrimental effects on cellular communication, leading to network disruptions within the central nervous system (CNS), such as those observed in patients with AD [4, 32]. Mounting evidence demonstrates that melatonin can protect synapses and dendritic spines from dysfunction in neurodegenerative diseases [33, 34]. Therefore, we analyzed PSD95 and spinophilin contents, which are markers of synapses and dendritic spines. PrP^106-126^ significantly suppressed the expression of PSD95 and spinophilin in N2a cells, and the reduction of these two proteins was prevented by melatonin. The primary site of energy consumption in neurons is localized at the synapse, where mitochondria are critical for both pre- and postsynaptic processes [35]. Significantly, we demonstrated that pretreatment with melatonin reduced synapses damage and nearly normalized mitochondria distribution in our prion model.

Mitochondrial morphology [36] and function reflect the status of mitochondrial homeostasis. Mitochondria produce ATP and ROS, but they are also susceptible to the adverse effects of ROS. Neuronal activity requires the consumption of large amounts of oxygen, and overproduction of ROS had been shown to be a major factor in almost all types of neurodegeneration [37]. In the present study, exposure to PrP^106-126^ led to mitochondrial dysfunction, as reflected by altered morphology, excessive ROS production, reduced ATP levels, and MMP disruption. During the progression of AD, APP, and Aβ accumulate in the mitochondrial membranes and cause structural and functional damage [38], reduce mitochondrial membrane potential, and compromise energy metabolism [39]. Melatonin protects neuronal cells from Aβ-mediated toxicity via its antioxidant and anti-amyloid effects [8, 29]. In a rat model of neuropathic pain, melatonin limited paclitaxel-induced mitochondrial dysfunction [40]. Similarly, our experiments showed that pretreatment with melatonin alleviated mitochondrial damage induced by PrP^106-126^. We revealed that mitochondrial damage induced by PrP^106-126^ is an important step in the neurotoxic effects. Our findings suggest that antioxidant capacity of melatonin may alleviate mitochondrial dysfunction in prion disease.

Mitochondrial dynamics imbalance occurs in most common neurodegenerative diseases, including AD, Parkinson's disease (PD), Huntington's disease (HD), and amyotrophic lateral sclerosis (ALS) [17]. There is evidence that altered mitochondrial dynamics cause cell injury and may contribute to the pathogenesis of AD [41]. Melatonin attenuates myocardial ischemia-reperfusion injury by activating the mitochondria fusion protein OPA1 to enhance mitochondrial fusion [42], and it also down-regulates expression of the mitochondria fission protein DRP1 to inhibit rotenone-induced SH-SY5Y cell death [43]. In our study, expression of DRP1 and OPA1 was disrupted by PrP^106-126^, while FIS1 and MFN1/2 remained unchanged. Pretreatment with melatonin inhibited the decrease of OPA1 and increase of DRP1 induced by PrP^106-126^. Inhibition of DRP1 by Mdivi-1 prevented PrP^106-126^-induced mitochondrial dysfunction including ATP levels. The combination of melatonin and Mdivi-1 increased ATP abundance more effectively than did Mdivi-1 alone. The change of ATP may be more closely related to OPA1 [44, 45]. These results suggest that DRP1 and OPA1 may play a role in the protective effect of melatonin in neurodegenerative diseases.

Taken together, our findings demonstrate neuroprotective effects of melatonin against prion-induced neural cell damage. We showed that pretreatment with melatonin inhibits mitochondrial-mediated apoptosis in the *in vitro* prion model. Melatonin protects synapses, mitochondrial morphology, and modulates mitochondrial dynamic proteins DRP1 and OPA1 from the detrimental effects of PrP^106-126^. Further studies are required to decipher the detailed mechanisms through which melatonin exerts these neuroprotective effects, and potential neuroprotection of melatonin in prion diseases should be further explored.

## MATERIALS AND METHODS

### Cell culture and treatment

Mouse neuroblastoma N2a cells were cultured in Dulbecco’s modified Eagle’s medium (DMEM) (Hyclone, Logan, UT, USA) supplemented with 10% (v/v) fetal bovine serum (Gibco, NY, USA) at 37 °C with 5% CO_2_ in a humid incubator. PrP^106-126^ peptide (KTNMKHMAGAAAAGAVVGGLG; >95% purity) was synthesized by Sangon Bio-Tech (Shanghai, China). The peptide was dissolved in 0.1 M phosphate-buffered saline (PBS) (Solarbio, Beijing, China) to a concentration of 1 mM and shaken at 4 °C for 24 h. All procedures were performed under sterile conditions. Experiments were conducted with a final peptide concentration of 100 μM.

Melatonin (Sigma-Aldrich, MO, USA) was dissolved in absolute ethanol and stored as a 50 mM stock solution at 4 °C. Mdivi-1 (MCE, Monmouth Junction, NJ, USA) was dissolved in DMSO.

### Cell viability assay

N2a cells were treated with melatonin at 0,1, 10 or 100 μM at 37 °C for 1 h before the addition of 100 μM PrP^106-126^ and further incubation for 24 h. Cell viability was determined using the Cell Counting Kit-8 assay kit (CCK-8; Beyotime, Shanghai, China). The CCK-8 solution was directly added to the cell culture medium before and incubated for 1 h at 37 °C in a 5% CO_2_ atmosphere. The absorbance at 450 nm was recorded using a microplate reader with a background control sample as the blank. The cell viability was expressed as percent of the untreated control.

### TUNEL assay

N2a cells were grown on coverslips at a density of 1 × 10^5^ cells per well in a 24-well plate and exposed to melatonin with or without PrP^106-126^ for 24 h. The cells were visualized using a confocal microscope (Olympus) and the One Step TUNEL Apoptosis Assay Kit (Beyotime, Shanghai, China).

### Determination of mitochondrial function

Reactive oxygen species in N2a cells was determined using 2′,7′-dichlorodihydrofluorescein diacetate (Beyotime, Shanghai, China). The mitochondrial membrane potential (MMP) was measured with a JC-1 Mitochondrial Membrane Potential Assay Kit (Beyotime, Shanghai, China). ATP was measured with an ATP Determination Kit (Beyotime, Shanghai, China). All procedures were performed following the manufacturer’s instructions.

### Mitochondrial isolation

Mitochondria of N2a cells were isolated with the Qproteome Mitochondria Isolation Kit (37612, Qiagen). The cells were washed in 0.1 M PBS, homogenized with a lysis buffer containing a protease inhibitor, and centrifuged at 1000 × *g* for 10 min to remove nuclear contaminants, cell debris, and intact cells. The supernatant was transferred to a clean 1.5-mL tube and centrifuged again at 6000 × *g* for 10 min at 4 °C. The supernatant containing the microsomal fraction was removed. All procedures were conducted at 4 °C.

### Transfection and infection

N2a cells were transfected with plasmids using Lipofectamine 3000 (Invitrogen). Plasmid DNA (0.5 μg) with 25 μL Opti-MEM medium was added to diluted Lipofectamine 3000, and the mixture was incubated for 10 minutes at room temperature. The DNA-lipid complex was added to 1 × 10^5^ adherent cells in a 24-well plate, after which the transfected cells were analyzed. The Mito-GFP construct was obtained from Clontech (Mountain View, CA USA), and pCAG-OPA1 was obtained from Vitalstar Biotechnology (Beijing, China).

### Western blotting

The cells were lysed with lysis buffer (Beyotime, Shanghai China) supplemented with a protease inhibitor solution (Beyotime) and centrifuged at 12 000 g for 10 min at 4 °C. Extracted proteins were separated on 10–15% sodium dodecyl sulfate polyacrylamide gels and transferred to nitrocellulose membranes. After blocking with 5% skim milk in Tris-buffered saline containing 0.1% Tween 20 (TBST) for 1 h at 37 °C, the membranes were incubated overnight with primary antibodies at 4 °C, washed with TBST, and then incubated with secondary antibodies. The western blot results were quantified by densitometric analysis using the Quantity One 4.6.9 software (Bio-Rad).

The following antibodies were used: anti-cleaved caspase-9 (9509T, CST), anti-cleaved caspase-3 (9664T, CST), anti-Bax (#2772, CST), anti-Bcl2 (#3498, CST), anti-cytochrome c (10093, Proteintech), VDAC rabbit mAb (4661, CST), anti-MFN1 (NBP1-71775, Novus Biologicals), DRP1 rabbit mAb (8570, CST), OPA1 rabbit mAb (80471, CST), anti-FIS1 (D122377-0025, BBI life sciences), anti-MFN2 (12186, Proteintech), anti-beta tubulin (10094, Proteintech), anti-PSD95 (20665, Proteintech), spinophilin rabbit mAb (14136, CST, Boston), HP-goat anti-mouse (ZB-2305, Zsbio, Beijing, China), HP-goat anti-rabbit (ZB-2301, Zsbio, Beijing, China), and Alexa Fluor 594 AffiniPure Goat Anti-Rabbit IgG (H+L) (33112ES60, Yeasen).

### Statistical analyses

All assays were repeated three times. The data were expressed as mean ± SD. Differences were analyzed by one-way ANOVA followed by Bonferroni’s post-hoc test using GraphPad Prism software version 5.0 (La Jolla, CA, USA) or ImageJ (National Institutes of Health, Bethesda, MD, USA). The threshold for significance was P < 0.05.
